# Development of a droplet digital PCR assay for the detection of BK polyomavirus

**DOI:** 10.1128/spectrum.01089-24

**Published:** 2024-10-14

**Authors:** Lu Ai, Yating Zhao, Chianru Tan, Lu Bai, Gang Huang, Ruizhi Wang, Hao Huang, Xuegao Yu, Yong Guo, Peisong Chen

**Affiliations:** 1 Department of Laboratory Medicine, The First Afﬁliated Hospital of Sun Yat-sen University, Guangzhou, Guangdong, China; 2 Department of Laboratory Medicine, Nansha Division of The First Affiliated Hospital, Sun Yat-sen University, Guangzhou, Guangdong, China; 3 School of Biomedical Engineering, Tsinghua University, Beijing, China; 4 Organ Transplant Center, The First Affiliated Hospital of Sun Yat-sen University, Guangzhou, China; Children's National Hospital, George Washington University, Washington, DC, USA

**Keywords:** BK polyomavirus, BK polyomavirus-associated nephropathy, droplet digital PCR

## Abstract

The objective of this study was to establish a more sensitive and specific diagnostic method for detecting plasma BK polyomavirus (BKPyV) DNA load in patients after renal transplantation using droplet digital polymerase chain reaction (ddPCR) and to validate the methodology. The linear range, lower limit of detection, accuracy, precision, and specificity of the detection system were evaluated by using the WHO BKPyV standard (7.2 log_10_ IU/mL) as a reference, in accordance with the relevant documents of the Clinical and Laboratory Standards Institute. Plasma samples were collected from 74 renal transplantation patients with urinary BKPyV-DNA levels exceeding 7 log_10_ copies/mL. Quantitative PCR (qPCR) and ddPCR were performed, and their diagnostic efficacy for BKPyV-DNA in the diagnosis of BK polyomavirus-associated nephropathy was evaluated using a receiver operating characteristic (ROC) curve. The coefficients of variation for the repeated detection of BKPyV standard DNA were 2.55 and 4.71 at concentrations of 6.2 and 3.2 log_10_ IU/mL, respectively. The linear range was 2.2–6.2 log_10_ IU/mL, and the lowest detection limit was 100 IU/mL. By utilizing histopathological examination of renal biopsy as the gold standard for BKPyV diagnosis, the area under the ROC curve of 74 post-transplantation plasma samples detected by the ddPCR system was found to be 0.875 (95% CI: 0.797–0.953, *P* < 0.01). The optimal threshold was 512.86 copies/mL, with a sensitivity of 90.0% and a specificity of 67.6%. In comparison, the area under the ROC curve for qPCR was 0.668 (95% CI: 0.583–0.752, *P* < 0.01), with an optimal threshold of 11,481.54 copies/mL, a sensitivity of 35.0%, and a specificity of 100.0%. Pairwise comparison (Delong test) of the ROC curves of the two systems showed a significant difference in the area under the curve, with a difference of 0.207 and a *P*-value <0.01. The BKPyV nucleic acid detection system, based on ddPCR, is appropriate for the regular monitoring of the BK polyomavirus, specifically in plasma samples containing low viral DNA loads while it provides the benefits of both absolute quantification and high sensitivity.

**IMPORTANCE:**

It was previously believed that droplet digital polymerase chain reaction had limitations, including high cost, limited throughput, and cumbersome operation, which hindered its widespread application in clinical practice. However, the current fully automated digital PCR platform, combined with streamlined operations, can detect 96 samples at once, and the entire process can be completed within an hour, laying a solid foundation for its extensive use.

## INTRODUCTION

BK polyomavirus (BKPyV) is a small DNA virus that was initially observed in 1971 when inclusion-bearing cells were found in the urine of a renal transplant recipient from Sudan who had ureteric stenosis (1). BKPyV is almost universally present in human populations and typically does not cause any symptoms in individuals with healthy immune systems (2). However, in individuals who have undergone a kidney transplant, BKPyV can reactivate and result in a condition known as BK polyomavirus-associated nephropathy (BKPyVAN), which can ultimately lead to the loss of the transplanted organ. As the use of potent immunosuppressive drugs has become more widespread and viral surveillance methods have improved, BKPyV has emerged as a significant cause of morbidity and mortality in kidney transplant recipients (3, 4). This poses a major challenge for medical professionals, as the condition often develops rapidly and aggressively, while effective antiviral treatments and prevention strategies are currently lacking.

Renal biopsy remains the gold standard for diagnosing BKPyVAN, despite being a time-consuming, invasive, and subjective procedure. It is worth noting that up to a third of renal biopsies fail to diagnose the condition due to the focal nature of the infection and the tendency for early disease to primarily affect the deeper collecting tubules within the kidney (5). Consequently, regular testing for plasma and urine BKPyV-DNA is crucial to detect early signs of nephropathy. According to “The Second International Consensus Guidelines on the Management of BK Polyomavirus in Kidney Transplantation,” screening should include monthly plasma BKPyV load tests until 9 months post-transplant, followed by testing every 3 months until 2 years post-transplant. If high levels of BKPyV (7 log_10_ copies/mL) are detected in urine, plasma BKPyV-DNA loads should be measured to guide treatment. Immunosuppression should be reduced if BKPyV-DNAemia exceeds 4 log_10_ copies/mL or if nephropathy is confirmed by biopsy (6). Although after kidney transplantation BKPyV appears first in urine compared to plasma, low concentrations of BKPyV in urine usually do not progress to graft failure. Early detection of BKPyV-DNA in the plasma of kidney transplant recipients can identify patients at risk of developing BKPyV, thus enabling prompt intervention and treatment. Hence, it is imperative to establish a more sensitive and accurate diagnostic approach to detect the BKPyV-DNA load in the plasma of kidney transplant patients, in order to enhance patient outcomes.

Currently, the most widely used method for the detection and quantification of BKPyV-DNA in clinical specimens is quantitative polymerase chain reaction (qPCR). However, one essential necessity for clinical PCR testing for viruses is the capacity to compare findings across different laboratories, notably when the data from a viral assay are utilized to establish clinical decision point levels across multiple institutions (4). Moreover, the reliability of qPCR in detecting low levels of BKPyV-DNA is questionable, particularly in the early stages of BKPyV (7). Our objective was to develop a more sensitive and specific diagnostic method to detect BKPyV-DNA load in the plasma of kidney transplant patients, utilizing digital droplet polymerase chain reaction (ddPCR) technology in order to address its limitations. DdPCR is a method to accurately measure the amount of specific DNA or RNA in a sample (8, 9). This technique divides the sample into numerous individual compartments, and then, it counts the number of compartments where the target nucleic acid is present (positive) and where it is not (negative) (10). It offers increased sensitivity, absolute quantification, improved precision, and robustness in the presence of PCR inhibitors, making it a powerful technique for accurate nucleic acid quantification (11, 12). Our hypothesis is that by employing ddPCR, we can achieve a more accurate and precise measurement of BKPyV-DNA load compared to qPCR, with a reduced detection limit and enhanced sensitivity, enabling the detection of even low levels of BKPyV-DNA.

## MATERIALS AND METHODS

### Sample collection and DNA extraction

A total of 74 plasma samples were collected from the First Affiliated Hospital of Sun Yat-sen University. The plasma samples were obtained from kidney transplant recipients whose BKPyV-DNA levels exceed 7 log_10_ copies/mL in their urine samples to assess the effectiveness of BKPyV-related nephropathy detection. All samples were anonymized and randomized, and the tester was blinded to the clinical test results. DNA extraction was carried out using the Daan gene DNA Extraction Kit DA0623 (Daan gene, China). The real-time qPCR and ddPCR techniques were employed to analyze the plasma samples, and the results were evaluated by means of receiver operating characteristic (ROC) curves.

### Designing of primers and TaqMan probes

The primers and TaqMan probe used to detect BKPyV small T antigen gene sequences were designed using the BKPyV genome available in the NCBI database as a reference. The sequences of the primers and probe were 5′-TTATTTGGACCCACCATTGC-3′ (forward primer), 5′-TGGATAGATTGCTACTGCATTG-3′ (reverse primer), and 5′-FAM-AGGTCTAAG+CCAAA+CCACT-BHQ1-3′ (probe, locked nucleic acid modification is represented by a plus sign +). The specificity and sensitivity of the primers and probes to BKPyV small T antigen gene sequences were ensured through testing using the standard BLAST application. Human RNase P gene was used as an internal amplification control gene.

### Automated ddPCR assay

**Fig 1:**
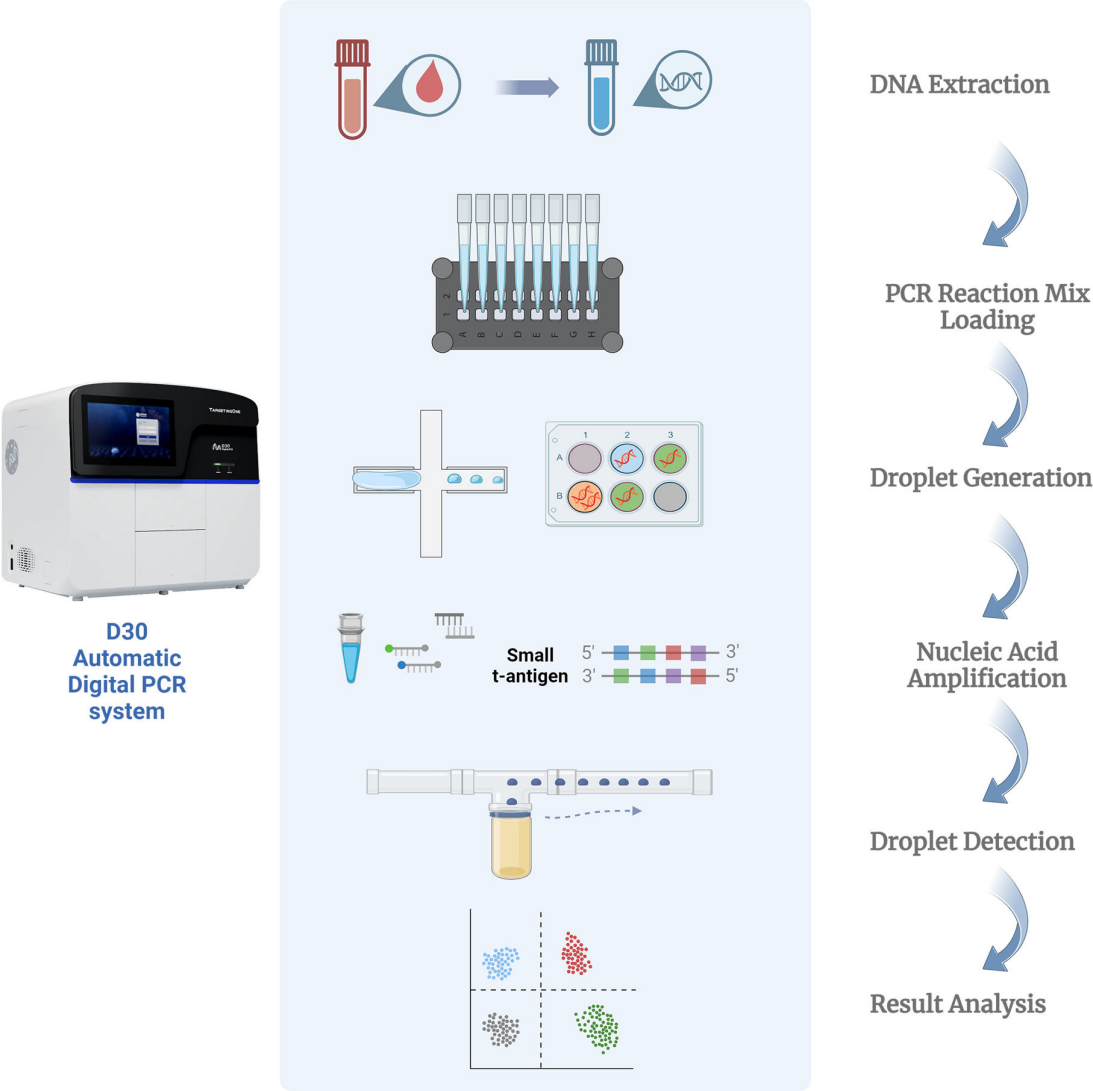
Procedural framework of the BK polyomavirus detection.

**Fig 2:**
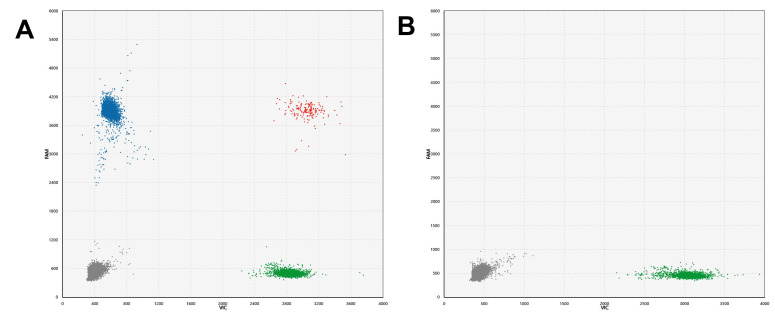
2-D cluster plot of droplet fluorescence for the reference gene and target gene in the ddPCR-based assay. Each droplet was sorted into one of the clusters: double negative (gray), reference gene positive (green), target gene positive (blue), and double positive (red).

The ddPCR was performed according to the manufacturer’s instructions using a fully automated digital PCR platform D30 (TargetingOne, Beijing, China). Briefly, a 30 µL ddPCR reaction mix was prepared for each sample, containing 7.5 µL of PCR mix, 7.5 µL of primer/probe mix, and 15 µL of DNA template. Thirty microliters of ddPCR reaction mix was added to the sample well of the integrated droplet microfluidic chip and sealed with 40 µL of sealing oil. The chip was covered with a rubber cap and placed into the chip loading box of the instrument. Then, water-in-oil droplets were generated in the instrument and stored in the reaction tube of the chip. The droplets were thermally cycled using a protocol of 95°C for 30 s, followed by 40 cycles of 94°C for 10 s and 57°C for 30 s. Finally, the droplets were driven by the detection oil to float up from the reaction tube to the microchannel of the chip, forming an orderly droplet queue and passing through the detection area in turn for fluorescence excitation and detection. By applying a fluorescence amplitude threshold at the highest point of the negative droplet cluster, it was possible to distinguish positive droplets containing amplified products from negative droplets. For analysis, reactions with more than 30,000 accepted droplets per well were employed. Using Poisson statistics, the exact initial copy number of target molecules was calculated and presented as copies/reaction of PCR, which was then calculated to reflect copies per milliliter of the sample. Figure 1 shows the procedural framework of the ddPCR-based assay for BKPyV detection. The procedural framework includes three steps: (i) DNA extraction using Daan gene DNA extraction kit DA0623 (Daan gene, China); (ii) preparation of reaction mix with the extracted DNA, primer/probe mix, and PCR reagent; and (iii) absolute quantification of the exact number of DNA copies of target and reference genes using ddPCR. Figure 2 shows the 2-D cluster plot of droplet fluorescence for the genes in the ddPCR-based assay. Each droplet was sorted into one of the clusters: double negative (gray), reference gene positive (green), target gene positive (blue), and double positive (red). Plot A corresponds to BKPyV positivity. Plot B corresponds to BKPyV negativity.

### qPCR assay

The detection of BKPyV by qPCR was performed using commercial kits equipped with TaqMan probes (SinoMD Quantitative detection kit for BKPyV nucleic acid, Beijing, China) on the Applied Biosystems 7500 Real Time System. The reaction system consisted of 25 µL, including 20 µL of the PCR reaction mixture and 5 µL of the DNA template. The amplification procedure was conducted as follows: 37°C for 2 min and 95°C for 3 min, followed by 40 cycles of 94°C for 15 s and 60°C for 35 s.

### Evaluation of ddPCR assay

**Fig 3:**
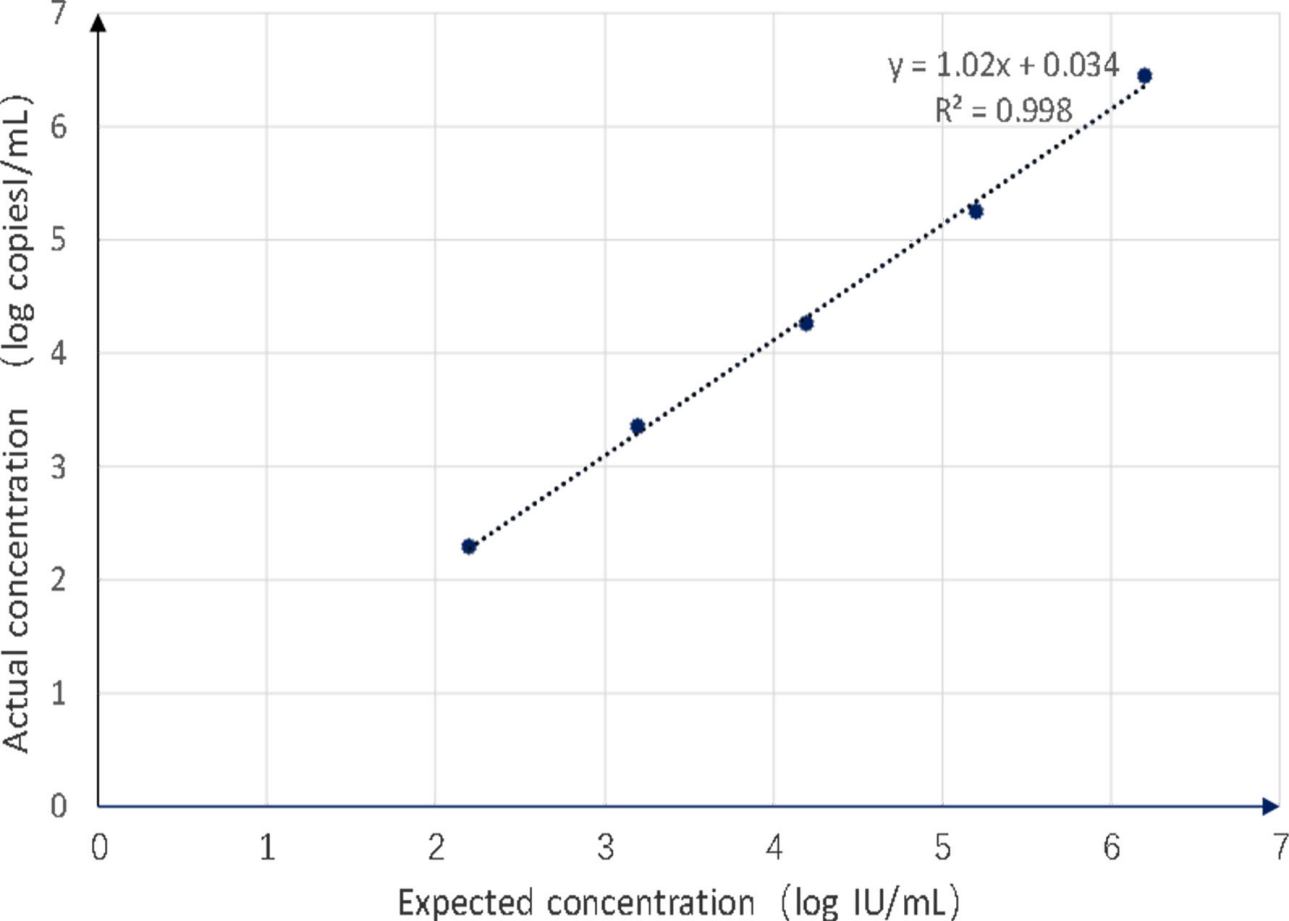
Conversions of copies/IU

The clinical study employed the WHO BKPyV standard sample (NIBSC code: 14/212) to assess the detection system’s linear range, lower limit of detection, accuracy, precision, and specificity. Based on the results of a worldwide collaborative study, this material has been assigned a concentration of 7.2 log_10_ international units (IU) per vial when reconstituted in 1 mL of nuclease-free water. After reconstitution, it is recommended that the international standard be diluted in the matrix frequently employed in the laboratory for the clinical diagnosis of BKPyV-DNA. We diluted the international standard with 1 mL of nuclease-free water, followed by serial 10-fold dilutions. The BKPyV verification panel then consisted of six concentrations (7.2, 6.2, 5.2, 4.2, 3.2, and 2.2 log_10_ IU/mL) of whole intact virus. Each concentration was extracted and subjected to quantitative real-time PCR (qPCR) and ddPCR. Conversions of copies/IU were calculated using logistic regression of the ddPCR results, *y*(copies) = 1.02*x*(IU) + 0.034 (Fig. 3).

#### Linearity and lower limit of detection

In line with the guidelines outlined by the Clinical and Laboratory Standards Institute, repeat testing was conducted for three wells of each concentration sample using ddPCR to confirm the assay’s linearity. Subsequently, the WHO BKPyV international standard was diluted to concentrations ranging from 100 to 12.5 IU/mL. Each concentration underwent testing in 25 replicates, in addition to a blank control, and the lower limit of detection (LLOD) is defined as the concentration at which 95% of positive samples are identified (>22 replicates), and a sample is considered positive if it shows the presence of at least one positive droplet.

#### Accuracy and precision

To assess the accuracy of the ddPCR assay, we tested diluted BKPyV standard samples at three concentrations: 5.2, 4.2, and 3.2 log_10_ IU/mL, with four replicates for each sample. We then calculated the bias between the detected concentration value and the theoretical value. Additionally, we conducted intra-assay precision tests in 12 replicates using two concentrations: 6.2 and 3.2 log_10_ IU/mL, to evaluate the precision of ddPCR.

#### Specificity

To evaluate the specificity of the ddPCR assay, other six common viruses (cytomegalovirus, Epstein–Barr virus, hepatitis B virus, hepatitis C virus, *Ureaplasma urealyticum*, and JC polyomavirus) were tested with the ddPCR assay.

### Data analysis

Normality was assessed using a Shapiro–Wilk test with a significance level of 0.05. The coefficient of variation [CV (%)] was calculated as the standard deviation (SD) divided by the mean. ROC curves were constructed based on histopathologic findings from renal biopsies, using sensitivity and specificity values obtained from qPCR and ddPCR results to validate the effectiveness of the detection system. The results were analyzed using the Delong test and correlation coefficients to assess the relationship between the two detection systems.

## RESULTS

### Linearity and lower limit of detection of ddPCR assay

**Fig 4:**
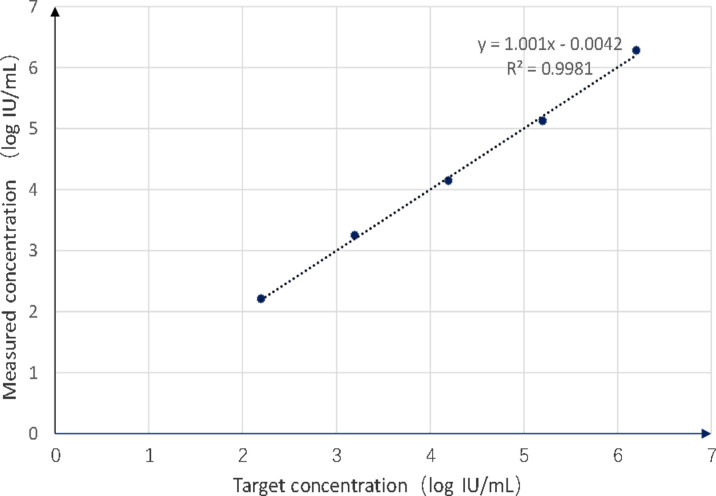
Standard curves of the WHO BKPyV standard constructed by ddPCR, and the quantification correlation was obtained by plotting the log assumed concentration against the log starting concentration.

The WHO BKPyV standard, serially diluted, demonstrated excellent linearity when using ddPCR assays over the linear range of 2.2–6.2 log_10_ IU/mL. In ddPCR, the standard curve demonstrated a strong linear correlation (*y* = *1.001x* -0.0042) with an *R*^2^ value of 0.9981 (Fig. 4). As illustrated in Table 1, the LLOD of ddPCR was determined to be 100 IU/mL. The high *R*² value supported the assay’s precision and robustness in quantifying the target, while the low LLOD indicated its ability to detect even low levels of the target DNA.

**TABLE 1:** Lower limit of detection of ddPCR assay

Concentration (IU/mL)	Hit rate	Positive/total
100	1.00	25/25
50	0.40	10/25
25	0.08	2/25
12.5	0.00	0/25
NTC	0.00	0/25

### Accuracy and precision of ddPCR assay

In the accuracy tests, the intra-assay CV ranged from 2.91% to 5.84% (Table 2). During the precision tests, the intra-assay CV of 6.2 and 3.2 log_10_ IU/mL were 2.55 and 4.71, respectively (Table 3). These results suggested that the ddPCR assay is reliable in terms of both accuracy and precision, meaning it can consistently produce accurate and reproducible measurements within the tested range.

**TABLE 2:** Accuracy analysis of ddPCR assay

Concentration (IU/mL)	Mean	SD	CV (%)
5.2 log_10_	5.19	0.17	3.29
4.2 log_10_	4.12	0.12	2.91
3.2 log_10_	3.17	0.18	5.84

**TABLE 3:** Precision analysis of ddPCR assay

Replicate	6.2 log_10_ IU/mL		3.2 log_10_ IU/mL	
	Concentration (IU/mL)	log_10_	Concentration (IU/mL)	log_10_
1	1,722,892.36	6.24	1,187.34	3.07
2	1,083,397.93	6.03	1,064.19	3.03
3	1,550,728.69	6.19	807.75	2.91
4	1,404,320.71	6.15	1,865.21	3.27
5	1,244,229.04	6.09	2,105.10	3.32
6	915,282.10	5.96	1,453.01	3.16
7	1,420,857.38	6.15	1,410.94	3.15
8	1,259,126.84	6.10	1,723.18	3.24
9	3,264,262.73	6.51	962.89	2.98
10	1,732,937.62	6.24	1,023.69	3.01
11	2,592,055.48	6.41	1,544.39	3.19
12	1,141,130.24	6.06	2,491.05	3.40
	Mean	6.18	Mean	3.14
	SD	0.16	SD	0.15
	CV (%)	2.55	CV (%)	4.71

### Specificity of ddPCR assay

For the specificity analysis, other pathogen (cytomegalovirus, Epstein–Barr virus, hepatitis B virus, hepatitis C virus, *Ureaplasma urealyticum*, and JC polyomavirus) tests were all negative, which indicated that this method exhibits high specificity for the detection of BKPyV.

### Clinical sample for screening

**Fig 5:**
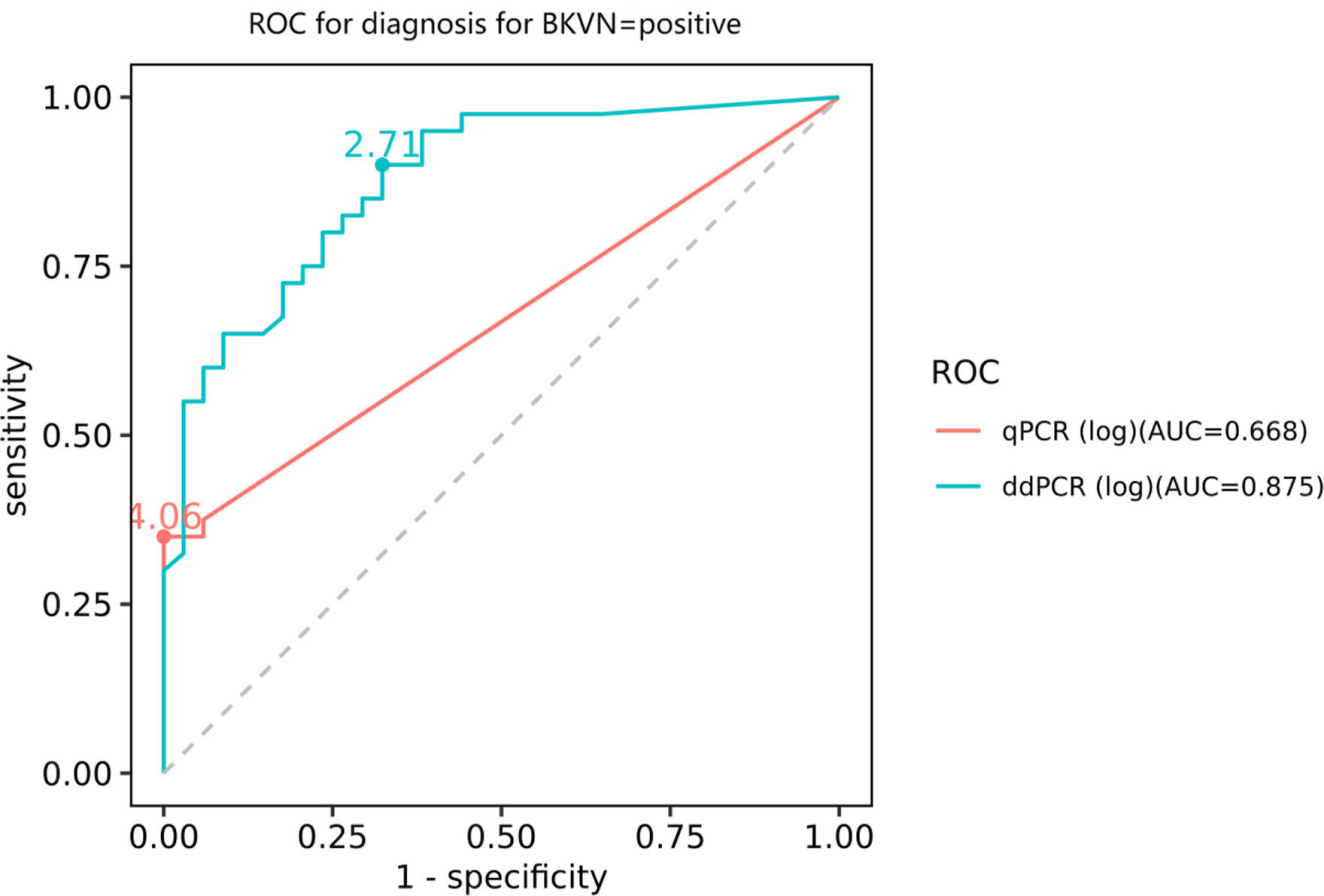
ROC curve for diagnosis for BKPyV positive with ddPCR and qPCR assays

To further evaluate the practicality of ddPCR, we simultaneously assessed 74 clinical samples using both ddPCR and qPCR. The positive cut-off values of qPCR and ddPCR were 5,000 and 118.58 copies/mL, respectively. As shown in (Table S1), 55 tested positive by ddPCR, while only 17 tested positive by qPCR. The discordance between ddPCR and qPCR results was as follows: 39 samples tested positive by ddPCR but negative by qPCR, and 1 sample tested positive by qPCR but negative by ddPCR. The lowest BKPyV load of 133 copies/mL was observed in a 63-year-old male with a renal allograft 6 months before. The area under the ROC curve for qPCR was 0.668 (95% CI: 0.583–0.752, *P* < 0.01), with an optimal threshold of 11,481.54 copies/mL, a sensitivity of 35.0%, and a specificity of 100.0%. For ddPCR, the area under the ROC curve was 0.875 (95% CI: 0.797–0.953, *P* < 0.01). The optimal threshold was 512.86 copies/mL, with a sensitivity of 90.0% and a specificity of 67.6%(Fig. 5). Pairwise comparison (Delong test) of the ROC curves of the two systems showed a significant difference in the area under the curve, with a difference of 0.207 and a *P*-value <0.01, indicating a significant difference in the diagnostic efficacy of the two assays for the diagnosis of BKPyVAN. These results indicated that ddPCR is significantly more sensitive and generally more effective than qPCR for detecting BKPyV, especially at lower viral loads.

## DISCUSSION

In recent years, the rates of BKPyV infection have been steadily increasing due to the widespread use of solid organ transplantation, new potent immunosuppressants, and innovative detection methods. BKPyVAN is a potentially life-threatening complication of kidney transplantation, with research estimating that up to 10% of kidney transplant recipients may develop this condition, ultimately leading to graft failure (1, 2, 13). Hence, the significance of early detection of BKPyV in the plasma of kidney transplant recipients cannot be overemphasized. It is important to note that BKPyVAN has been reported in a pediatric patient with a viral load as low as 200 copies/mL plasma (14). This suggests that even low viral loads in plasma should be closely monitored. As a third-generation PCR technique, ddPCR has been widely used for the detection and quantitative analysis of a diverse range of viruses (15). Due to its enhanced detection sensitivity and high tolerance for many PCR inhibitors, ddPCR is more suitable for the detection of low-level virus genomic copies (16, 17).

In this study, a novel ddPCR method for the detection and quantification of BKPyV was established. This method exhibited high sensitivity and accuracy, with low intra-assay coefficients of variation (<5.0%). These results indicate that the ddPCR method can provide accurate and reproducible detection results for BKPyVAN diagnosis. Among 74 clinical samples tested by both ddPCR and qPCR, 39 samples yielded positive results by ddPCR but negative by qPCR. Subsequent kidney biopsy pathology confirmed the presence of BKPyV, suggesting that the ddPCR method has a higher detection rate for BKPyV compared to qPCR. Furthermore, the quantitative results of ddPCR indicated that 30 were below the detection limit of qPCR, while 7 exceeded the detection limit and were still not detected by qPCR. We speculated that the issue may be related to inhibitors or excess background DNA, which can impact amplification efficiency and alter the relationship between Ct and target number (11, 12). In ddPCR, partitioning enriches the target from the background, which improves amplification efficiency and tolerance to inhibitors.

The ROC analysis results indicated that the optimal threshold for ddPCR was significantly lower than that for qPCR. As a result, while ddPCR offers superior sensitivity, it comes at the cost of lower specificity compared to qPCR. The lower value could be attributed to the incomplete coverage of all populations in the selected sample. On the other hand, the recent guidelines on the management of BKPyV in kidney transplantation suggested that persistent levels between 3 and 4 log_10_ copies/mL indicate probable nephropathy, even without biopsy confirmation (6), underscoring the necessity of setting a cutoff below 4 log_10_ copies/mL to prompt a review of immunosuppression. Some studies suggested that a certain threshold of viruria must be reached before the virus becomes apparent in the plasma. This threshold seems to vary from patient to patient. Thus, prior to the onset of viremia, a notable increase in the urinary viral load should prompt the transplant clinician to take notice and consider adjusting the immunosuppressive regimen (14). Owing to its high sensitivity, screening with plasma samples alone has proven to be an effective method for patients, eliminating the need for simultaneous screening with both plasma and urine samples and aligning with recent guidelines (6). The superior diagnostic performance of ddPCR (as indicated by the higher AUC and greater sensitivity) makes it a better choice for the early detection and monitoring of BKPyV infection in clinical settings.

An additional advantage of the ddPCR assay is its ability to achieve absolute quantification without the need to establish a standard curve, enabling the exchange of results among different laboratories.(18) In contrast, the qPCR assay can only achieve quantitative detection using a calibration curve produced from serially diluted templates, with the copy number in samples being dependent on the qPCR Ct values from the standard curve. It was previously believed that ddPCR had limitations, including high cost, limited throughput, and cumbersome operation, which hindered its widespread application in clinical practice. However, the current fully automated digital PCR platform, combined with streamlined operations, can detect 96 samples at once, and the entire process can be completed within an hour, laying a solid foundation for its extensive use. Moreover, the on-demand testing mode of ddPCR, as opposed to batch-processing dozens of samples simultaneously of qPCR, has accelerated the testing process, enabling patients to swiftly receive results within hours, thereby enhancing the speed of diagnosis.

This study had several limitations. First, the sample size was relatively small, necessitating further multi-center research with a larger cohort. Second, repeated testing and a third assay for plasma samples with discrepant results should be conducted to improve accuracy. It is clear that BKPyV continues to present a significant challenge in renal transplantation.(19, 20) Further research is necessary to determine why clinical disease occurs in certain patients and understand the significant variations in the clinical manifestations of the disease. In our article, we have developed and evaluated a sensitive and reliable droplet digital PCR assay for the detection of BKPYV in clinical samples. Additionally, we will continue to refine the ddPCR assay to improve its suitability for the early detection of BKPyV, thereby aiding in the prevention and control of virus spread.

## Data Availability

The data are available from the corresponding author on reasonable request.

## SUPPLEMENTAL MATERIAL

Table S1 spectrum.01089-24-s0001.docx The results of 74 clinical samples tested by qPCR and ddPCR.

ASM does not own the copyrights to Supplemental Material that may be linked to, or accessed through, an article. The authors have granted ASM a non-exclusive, world-wide license to publish the Supplemental Material files. Please contact the corresponding author directly for reuse.

## References

[1] Gardner SD, Field AM, Coleman DV, Hulme B. 1971. New human papovavirus (B.K.) isolated from urine after renal transplantation. Lancet 1:1253–1257. doi:10.1016/s0140-6736(71)91776-4 4104714

[2] Park WY, Kang SS, Jin K, Park SB, Choe M, Han S. 2018. Long-term prognosis of BK virus-associated nephropathy in kidney transplant recipients. Kidney Res Clin Pract 37:167–173. doi:10.23876/j.krcp.2018.37.2.167 29971212 PMC6027809

[3] Vasudev B, Hariharan S, Hussain SA, Zhu YR, Bresnahan BA, Cohen EP. 2005. BK virus nephritis: risk factors, timing, and outcome in renal transplant recipients. Kidney Int 68:1834–1839. doi:10.1111/j.1523-1755.2005.00602.x 16164661

[4] Knowles WA, Pipkin P, Andrews N, Vyse A, Minor P, Brown DWG, Miller E. 2003. Population-based study of antibody to the human polyomaviruses BKV and JCV and the simian polyomavirus SV40. J Med Virol 71:115–123. doi:10.1002/jmv.10450 12858417

[5] Drachenberg CB, Papadimitriou JC, Hirsch HH, Wali R, Crowder C, Nogueira J, Cangro CB, Mendley S, Mian A, Ramos E. 2004. Histological patterns of polyomavirus nephropathy: correlation with graft outcome and viral load. Am J Transplant 4:2082–2092. doi:10.1046/j.1600-6143.2004.00603.x 15575913

[6] Kotton CN, Kamar N, Wojciechowski D, Eder M, Hopfer H, Randhawa P, Sester M, Comoli P, Tedesco Silva H, Knoll G, Brennan DC, Trofe-Clark J, Pape L, Axelrod D, Kiberd B, Wong G, Hirsch HH, Transplantation Society International BK Polyomavirus Consensus Group. 2024. The second international consensus guidelines on the management of BK polyomavirus in kidney transplantation. Transplantation 108:1834–1866. doi:10.1097/TP.0000000000004976 38605438 PMC11335089

[7] Hirsch HH, Babel N, Comoli P, Friman V, Ginevri F, Jardine A, Lautenschlager I, Legendre C, Midtvedt K, Muñoz P, Randhawa P, Rinaldo CH, Wieszek A, ESCMID Study Group of Infection in Compromised Hosts. 2014. European perspective on human polyomavirus infection, replication and disease in solid organ transplantation. Clin Microbiol Infect 20 Suppl 7:74–88. doi:10.1111/1469-0691.12538 24476010

[8] Liu B, Panda D, Mendez-Rios JD, Ganesan S, Wyatt LS, Moss B. 2018. Identification of poxvirus genome uncoating and DNA replication factors with mutually redundant roles. J Virol 92:e02152-17. doi:10.1128/JVI.02152-17 29343579 PMC5972866

[9] Liu BM, Rakhmanina NY, Yang Z, Bukrinsky MI. 2024. Mpox (Monkeypox) virus and its co-infection with HIV, sexually transmitted infections, or bacterial superinfections: double whammy or a new prime culprit? Viruses 16:784. doi:10.3390/v16050784 38793665 PMC11125633

[10] Vogelstein B, Kinzler KW. 1999. Digital PCR. Proc Natl Acad Sci U S A 96:9236–9241. doi:10.1073/pnas.96.16.9236 10430926 PMC17763

[11] Basu AS. 2017. Digital assays part I: partitioning statistics and digital PCR. SLAS Technol 22:369–386. doi:10.1177/2472630317705680 28448765

[12] Salipante SJ, Jerome KR. 2020. Digital PCR-an emerging technology with broad applications in microbiology. Clin Chem 66:117–123. doi:10.1373/clinchem.2019.304048 31704712

[13] Chong S, Antoni M, Macdonald A, Reeves M, Harber M, Magee CN. 2019. BK virus: current understanding of pathogenicity and clinical disease in transplantation. Rev Med Virol 29:e2044. doi:10.1002/rmv.2044 30958614

[14] Herman J, Van Ranst M, Snoeck R, Beuselinck K, Lerut E, Van Damme-Lombaerts R. 2004. Polyomavirus infection in pediatric renal transplant recipients: evaluation using a quantitative real-time PCR technique. Pediatr Transplant 8:485–492. doi:10.1111/j.1399-3046.2004.00211.x 15367285

[15] Deiana M, Mori A, Piubelli C, Scarso S, Favarato M, Pomari E. 2020. Assessment of the direct quantitation of SARS-CoV-2 by droplet digital PCR. Sci Rep 10:18764. doi:10.1038/s41598-020-75958-x 33127953 PMC7599326

[16] Suo T, Liu X, Feng J, Guo M, Hu W, Guo D, Ullah H, Yang Y, Zhang Q, Wang X, Sajid M, Huang Z, Deng L, Chen T, Liu F, Xu K, Liu Y, Zhang Q, Liu Y, Xiong Y, Chen G, Lan K, Chen Y. 2020. ddPCR: a more accurate tool for SARS-CoV-2 detection in low viral load specimens. Emerg Microbes Infect 9:1259–1268. doi:10.1080/22221751.2020.1772678 32438868 PMC7448897

[17] Frías M, Rivero-Juárez A, Téllez F, Palacios R, Jiménez-Arranz Á, Pineda JA, Merino D, Gómez-Vidal MA, Pérez-Camacho I, Camacho Á, Rivero A. 2019. Evaluation of hepatitis C viral RNA persistence in HIV-infected patients with long-term sustained virological response by droplet digital PCR. Sci Rep 9:12507. doi:10.1038/s41598-019-48966-9 31467339 PMC6715682

[18] Pinheiro LB, Coleman VA, Hindson CM, Herrmann J, Hindson BJ, Bhat S, Emslie KR. 2012. Evaluation of a droplet digital polymerase chain reaction format for DNA copy number quantification. Anal Chem 84:1003–1011. doi:10.1021/ac202578x 22122760 PMC3260738

[19] Egli A, Infanti L, Dumoulin A, Buser A, Samaridis J, Stebler C, Gosert R, Hirsch HH. 2009. Prevalence of polyomavirus BK and JC infection and replication in 400 healthy blood donors. J Infect Dis 199:837–846. doi:10.1086/597126 19434930

[20] Ginevri F, Azzi A, Hirsch HH, Basso S, Fontana I, Cioni M, Bodaghi S, Salotti V, Rinieri A, Botti G, Perfumo F, Locatelli F, Comoli P. 2007. Prospective monitoring of polyomavirus BK replication and impact of pre-emptive intervention in pediatric kidney recipients. Am J Transplant 7:2727–2735. doi:10.1111/j.1600-6143.2007.01984.x 17908275

