# Supplementary material for: Development of a droplet digital PCR assay for the detection of BK polyomavirus
Source: Microbiol Spectr. 2024 Oct 14;12(11):e01089-24. doi: 10.1128/spectrum.01089-24 (PMC11536987; doi:10.1128/spectrum.01089-24)
Supplement: Table S1 — The results of 74 clinical samples tested by qPCR and ddPCR. [file spectrum.01089-24-s0001.docx]

**Development of a droplet digital PCR assay for detection of**

**BK polyomavirus**

**Supplemental Data**

**Table S1 The results of 74 clinical samples tested by qPCR and ddPCR**

| Sample No. | qPCR  (copies/ml) | qPCR  (log10) | ddPCR  (copies/ml) | ddPCR  (log10) |
| --- | --- | --- | --- | --- |
| 1 | 0.00E+00 | 0.00 | 0.00E+00 | 0.00 |
| 2 | 0.00E+00 | 0.00 | 0.00E+00 | 0.00 |
| 3 | 0.00E+00 | 0.00 | 0.00E+00 | 0.00 |
| 4 | 0.00E+00 | 0.00 | 0.00E+00 | 0.00 |
| 5 | 0.00E+00 | 0.00 | 0.00E+00 | 0.00 |
| 6 | 0.00E+00 | 0.00 | 0.00E+00 | 0.00 |
| 7 | 9.32E+03 | 3.97 | 2.68E+03 | 3.43 |
| 8 | 4.95E+04 | 4.69 | 2.61E+04 | 4.42 |
| 9 | 0.00E+00 | 0.00 | 0.00E+00 | 0.00 |
| 10 | 0.00E+00 | 0.00 | 2.87E+04 | 4.46 |
| 11 | 2.38E+04 | 4.38 | 2.85E+02 | 2.45 |
| 12 | 0.00E+00 | 0.00 | 2.43E+04 | 4.39 |
| 13 | 8.81E+03 | 3.95 | 2.68E+03 | 3.43 |
| 14 | 5.68E+04 | 4.75 | 2.87E+04 | 4.46 |
| 15 | 0.00E+00 | 0.00 | 9.11E+03 | 3.96 |
| 16 | 2.13E+05 | 5.33 | 0.00E+00 | 0.00 |
| 17 | 2.98E+04 | 4.47 | 9.11E+03 | 3.96 |
| 18 | 0.00E+00 | 0.00 | 7.48E+03 | 3.87 |
| 19 | 0.00E+00 | 0.00 | 1.73E+04 | 4.24 |
| 20 | 0.00E+00 | 0.00 | 0.00E+00 | 0.00 |
| 21 | 1.41E+04 | 4.15 | 7.95E+04 | 4.90 |
| 22 | 5.64E+05 | 5.75 | 6.29E+05 | 5.80 |
| 23 | 1.44E+04 | 4.16 | 1.15E+04 | 4.06 |
| 24 | 0.00E+00 | 0.00 | 5.29E+03 | 3.72 |
| 25 | 1.71E+04 | 4.23 | 1.84E+04 | 4.26 |
| 26 | 1.71E+04 | 4.23 | 7.00E+04 | 4.85 |
| 27 | 1.91E+05 | 5.28 | 1.20E+05 | 5.08 |
| 28 | 9.03E+03 | 3.96 | 5.04E+02 | 2.70 |
| 29 | 3.38E+05 | 5.53 | 1.19E+06 | 6.07 |
| 30 | 8.21E+04 | 4.91 | 1.64E+06 | 6.22 |
| 31 | 0.00E+00 | 0.00 | 0.00E+00 | 0.00 |
| 32 | 1.71E+04 | 4.23 | 1.54E+05 | 5.19 |
| 33 | 0.00E+00 | 0.00 | 3.86E+06 | 6.59 |
| 34 | 0.00E+00 | 0.00 | 5.47E+02 | 2.74 |
| 35 | 0.00E+00 | 0.00 | 4.37E+02 | 2.64 |
| 36 | 0.00E+00 | 0.00 | 1.22E+03 | 3.09 |
| 37 | 0.00E+00 | 0.00 | 9.93E+02 | 3.00 |
| 38 | 0.00E+00 | 0.00 | 8.40E+01 | 1.92 |
| 39 | 0.00E+00 | 0.00 | 6.62E+02 | 2.82 |
| 40 | 0.00E+00 | 0.00 | 5.48E+04 | 4.74 |
| 41 | 0.00E+00 | 0.00 | 0.00E+00 | 0.00 |
| 42 | 0.00E+00 | 0.00 | 2.33E+02 | 2.37 |
| 43 | 0.00E+00 | 0.00 | 1.59E+02 | 2.20 |
| 44 | 0.00E+00 | 0.00 | 0.00E+00 | 0.00 |
| 45 | 0.00E+00 | 0.00 | 2.63E+03 | 3.42 |
| 46 | 0.00E+00 | 0.00 | 5.48E+03 | 3.74 |
| 47 | 0.00E+00 | 0.00 | 5.36E+06 | 6.73 |
| 48 | 0.00E+00 | 0.00 | 1.33E+02 | 2.12 |
| 49 | 0.00E+00 | 0.00 | 5.23E+02 | 2.72 |
| 50 | 0.00E+00 | 0.00 | 1.62E+03 | 3.21 |
| 51 | 0.00E+00 | 0.00 | 6.23E+02 | 2.79 |
| 52 | 0.00E+00 | 0.00 | 3.97E+01 | 0.00 |
| 53 | 0.00E+00 | 0.00 | 6.84E+02 | 2.83 |
| 54 | 0.00E+00 | 0.00 | 5.04E+02 | 2.70 |
| 55 | 0.00E+00 | 0.00 | 7.37E+06 | 6.87 |
| 56 | 0.00E+00 | 0.00 | 2.59E+03 | 3.41 |
| 57 | 0.00E+00 | 0.00 | 3.83E+03 | 3.58 |
| 58 | 0.00E+00 | 0.00 | 6.46E+02 | 2.81 |
| 59 | 0.00E+00 | 0.00 | 3.02E+03 | 3.48 |
| 60 | 0.00E+00 | 0.00 | 1.03E+02 | 2.01 |
| 61 | 0.00E+00 | 0.00 | 7.82E+03 | 3.89 |
| 62 | 0.00E+00 | 0.00 | 8.40E+01 | 1.92 |
| 63 | 0.00E+00 | 0.00 | 7.55E+03 | 3.88 |
| 64 | 0.00E+00 | 0.00 | 6.79E+03 | 3.83 |
| 65 | 0.00E+00 | 0.00 | 4.81E+03 | 3.68 |
| 66 | 0.00E+00 | 0.00 | 9.80E+01 | 1.99 |
| 67 | 0.00E+00 | 0.00 | 1.66E+03 | 3.22 |
| 68 | 0.00E+00 | 0.00 | 1.44E+03 | 3.16 |
| 69 | 0.00E+00 | 0.00 | 3.50E+01 | 1.54 |
| 70 | 0.00E+00 | 0.00 | 5.88E+02 | 2.77 |
| 71 | 0.00E+00 | 0.00 | 2.26E+02 | 2.35 |
| 72 | 0.00E+00 | 0.00 | 1.40E+04 | 4.15 |
| 73 | 0.00E+00 | 0.00 | 1.43E+05 | 5.15 |
| 74 | 0.00E+00 | 0.00 | 8.40E+01 | 1.92 |

The color-filled cells indicate positive results.
